# TAILORED CARE FOR TERMINALLY-ILL OLDER ADULTS: INTEGRATED COMMUNITY-BASED END-OF-LIFE SUPPORT TEAM MODEL (ICEST)

**DOI:** 10.1093/geroni/igae098.3064

**Published:** 2024-12-31

**Authors:** Anna Yan Zhang, Estelle Xun Fang, Gloria Ka Ming Chun

**Affiliations:** The University of Hong Kong, Hong Kong, China (People’s Republic); The University of Hong Kong, Hong Kong, China (People’s Republic); The University of Hong Kong, Hong Kong, China (People’s Republic)

## Abstract

**Background:**

The integrated community-based end-of-life support team (ICEST) tailored the physical-psychosocial-practical care for community-dwelling terminally ill patients and enhanced the capacity of family caregiving through ongoing assessments and seamless interdisciplinary cooperation. The study evaluated the deceased patients’ first three-month care profiles and outcomes.

**Methods:**

An intervention study with three-time-point repeated measures. Public hospitals identified and referred eligible older adults (age ≥ 60, life expectancy ≤12 months) to receive ICEST services. Patients’ clinical symptoms were measured at baseline, one month, and three months after, and the care they received was recorded. Deceased cases (n=756) were extracted for analysis. Repeated ANOVA was used to analyze the changes in care profiles and clinical outcomes using SPSS 27.0.

**Results:**

The ICEST could significantly reduce patients’ physical and psycho-social-spiritual symptoms. Except for patients with a short survival period (≤3 months), they experienced a sharp worsening of pain (F (3,188)=2.82,p<.05) and weakness (F (3,188)=5.32,p<.01) and drowsiness remained consistently high (F (3,190)=6.29,p<.001). Depression levels of patients with a longer survival period (>12 months) decreased more after 1st month (F(3,168)=2.68,p<.05). The highest input was in psychosocial care. No significant change was found in its input or physical care, and practical support increased after the 1st month. Psychosocial care for patients with shorter survival periods (≤3 months) was higher initially and decreased later, whereas others showed the opposite trend.

**Conclusion:**

Various care profiles indicated the moment-by-moment changes in care demands among community-dwelling terminally ill patients. Timely assessments and corresponding adjustments of interventions to address real-time needs are essential.

